# Effects of dyadic-based physical activity intervention on cancer-related fatigue among cancer survivors: A scoping review

**DOI:** 10.3389/fpsyg.2023.1102019

**Published:** 2023-01-25

**Authors:** Dongyu Song, Yuzhou Liu, Claudia K. Y. Lai, Yuli Li

**Affiliations:** ^1^School of Nursing and Rehabilitation, Cheeloo College of Medicine, Shandong University, Jinan, Shandong, China; ^2^School of Nursing, The Hong Kong Polytechnic University, Kowloon, Hong Kong SAR, China

**Keywords:** fatigue, physical activity, dyadic intervention, cancer, review

## Abstract

**Objective:**

Cancer-related fatigue is one of the most common adverse reactions to cancer survivors, which has a significant impact on the daily life. As a traumatic event, cancer not only brings great physical and mental harm to patients, but also poses a threat to the physical and psychological health of caregivers. Current studies have shown that physical activity improves cancer-related fatigue in cancer survivors. And studies have suggested that dyadic interventions are more effective in improving patient outcomes and may also provide some benefits to caregivers. But the literature on the effects of dyadic-based physical activity on improving cancer-related fatigue has not been synthesized. This scoping review described the scope and impact of studies on cancer-related fatigue with dyadic-based physical activity interventions.

**Methods:**

Six databases which is PubMed, Cochrane Library, Web of Science, Embase, CINAHL and Medline were searched for all studies of dyadic-based physical activity interventions with outcome measures including cancer-related fatigue published since the inception of the databases through May 2022. The search strategy was developed based on PICO principles.

**Results:**

This article includes 6 pre and post-test designs and 2 randomized controlled trial design. The majority of participants were survivors with breast and lung cancer. The overall results showed that the effectiveness of dyadic-based physical activity interventions in improving cancer-related fatigue was unsatisfactory.

**Conclusions:**

This scoping review suggests that current dyadic-based physical activity interventions are not well-researched among cancer survivors. In the future, more high-quality studies with more sophisticated and rigorous interventions are needed.

## 1. Introduction

With the continuous advancement of medical diagnosis and treatment technology, the popularity of early screening and comprehensive cancer care, cancer survival rate has increased and survival with cancer has become a common phenomenon but various adverse effects associated with it are still worthy of concern. Cancer-related fatigue is one of the most commonly reported discomfort in cancer survivors (Frikkel et al., 2020). The National Comprehensive Cancer Network (NCCN) Clinical Practice Guidelines in Oncology defines cancer-related fatigue (CRF) as a multidimensional structural symptom that includes physical, cognitive, and emotional pain, is related to cancer treatment, and affects the survivor's daily functional activities (NCCN, 2018). Unlike general fatigue, it is often sudden, rapid, lasts for months or even years and cannot be relieved by rest (Bower et al., 2000; Yang et al., 2019). In most studies, 30–60% of cancer survivors experienced CRF during treatment (Bower, 2014), which significantly reduced the quality of daily life (Yang et al., 2019) and shortened survival (Quinten et al., 2011). Related studies have found that when compared with survivors with low fatigue trajectory, survivors with high fatigue trajectory were more often associated with anxiety, depression and sleep disorders (Bower et al., 2018; Dean, 2022). Therefore, it is necessary to take appropriate measures and interventions to reduce CRF for cancer survivors.

Several pharmacological and non-pharmacological measures have been shown to alleviate CRF, such as psychological guidance, acupuncture, etc. (Wu et al., 2019; Thong et al., 2020; Haussmann et al., 2022). In 2021, NCCN suggest that physical activity can be used to effectively manage CRF symptoms in cancer survivors (Network NCC, 2021), and physical activity is a first level evidence based intervention for CRF (Mitchell et al., 2007). A meta-analysis showed that regular physical activity reduced fatigue and increased survivors self-reported physical function (Juvet et al., 2017). Also, a yoga therapy program was found to be effective in treating CRF in a national multicenter randomized controlled trial (Lin et al., 2019). However, among cancer survivors, their physical activity compliance is low due to limited knowledge of the disease, lack of social support, and poor mental status (Tang et al., 2017; Sansano-Nadal et al., 2019).

Relevant studies have shown that dyadic intervention considers the influence of social and environmental factors on physical activity and can improve the compliance and efficacy of physical activity intervention by increasing social support and strengthening social relations (Cobb et al., 2016; Winters-Stone et al., 2016; Ellis et al., 2017). In addition, based on previous use of the actor-partner interdependence model to explore the cancer survivors-caregiver relationship, the researchers found that the adverse effects experienced by cancer survivors can spread from the survivors to their caregivers (Saita et al., 2022), and caregivers of cancer survivors may experience the same or even more symptoms as survivors (Milbury et al., 2019). These adverse effects can in turn affect their engagement in caregiving activities. As such, it becomes particularly important to examine the effects of dyadic-based physical activity interventions for survivors with cancer and their family caregivers.

Dyadic intervention focuses on the interdependence and influence between the survivors and caregivers, and treats the two parties as a “unit” for overall intervention (Hu et al., 2019). Systematic evaluation shows that dyadic intervention can improve the quality of life, mental state, stress, communication and cancer-related health status of survivors and their caregivers (Brandão et al., 2014; Stefǎnut et al., 2020). The existing forms of dyadic interventions mainly use psychoeducation, positive thinking, yoga, and Tai chi etc. (Sharma et al., 2021). Concerning the efficacy of dyadic-based physical activity interventions on CRF, there were fewer studies and the evidence of their effectiveness is insufficient. To the best of our knowledge, there is no comprehensive systematic review in the literature summarizing the effects of dyadic-based physical activity interventions on CRF outcomes. Therefore, the purpose of this paper is to provide a scoping review of the status and effect of dyadic-based physical activity interventions to improve CRF.

## 2. Method

Arksey and O'Malley (2005) framework was used to guide the completion of this review. The framework is built from the following five distinct steps: (i) identifying the research questions; (ii) identifying relevant studies; (iii) selecting studies; (iv) charting the data; and (v) collating, summarizing, and reporting the results of the selected studies. To ensure appropriate rigor and transparency in reporting, the reporting of study results followed the Preferred Reporting Items for Systematic Reviews and Extensions for Meta-Analyses (PRISMA-ScR) reporting guidelines (Tricco et al., 2018).

### 2.1. Identifying the research questions

Before conducting the literature search, we identified the following specific research issues: (i) What was the current status of existing research on dyadic-based physical activity interventions for improving CRF among cancer survivors? (ii) How effective was dyadic-based physical activity interventions described in the literature in improving CRF?

### 2.2. Identifying relevant studies

Six databases (PubMed, Cochrane Library, Web of Science, Embase, CINAHL, Medline) were searched from the inception of the database to May 2022. Free text terms and relevant subject headings (i.e., MeSH, EMTREE) for “neoplasms” (cancer, tumor), “cancer-related fatigue” (CRF, fatigue), and “dyad^*^” (couple, family) were used. These terms were also combined with implementation study terms (e.g., “intervention,” “program,” etc.) using the Boolean logic operators (OR, AND). The reference list of relevant articles was also hand searched for eligible studies that met the inclusion criteria.

### 2.3. Selecting studies

Prior to article selection, the research team discussed the development of inclusion criteria based on our research questions. The inclusion criteria developed included: (i) adult survivors with a cancer diagnosis regardless of cancer site, stage, or active treatment modality; (ii) CRF was included as one of the outcome measures; (iii) the type of intervention was dyadic-based physical activity, defined as cancer survivors and other partners physical activity together; and (iv) articles published in English and available in full.

The screening process was conducted using the PRISMA-ScR scoping review methodology (Tricco et al., 2018). After completing the relevant searches from the six databases, we used EndNote 27 software to identify and remove duplicate papers. Screening was conducted independently by SDY and LYZ. First, the titles and abstracts were read, and any differences of opinion was resolved through discussion until a consensus to exclude irrelevant studies could be reached. Then, the full text of all identified papers was read for a second round of screening. In case of disagreement, the same process of deliberation until a consensus could be reached among the team was repeated. Throughout the processes, there was no need to bring in an external expert to resolve disagreements.

### 2.4. Charting the data

SDY and LYZ used data charts to extract information from each article included. The following data were extracted and input it into Tables 1, 2: (i) research information (i.e., author, year of publication); (ii) sample characteristics (i.e., cancer type, stage, age, gender, relationship between the subjects); (iii) research characteristics (design, research tools used, intervention frequency and duration); and (iv) research results (CRF).

**Table 1:** Characteristics of study participants.

**References**	**Survivors**				**Partners**		**Relationship, *n* (%)**
	**Type of cancer**	**Disease stage**, ***n*** **(%)**	**Age (** ***N*** **)**	**Gender (female)**, ***n*** **(%)**	**Age (** ***N*** **)**	**Gender (female)**, ***n*** **(%)**	
Milbury et al. (2015a)	Non-small-cell lung cancer	IIIA: 5 (55.6) IIIB: 4 (44.4)	62.16 ± 14.03 (*N* = 9)	4 (44.4)	58.95 ± 15.67 (*N* = 9)	6 (66.7)	Spouse: 6 (66.7); Other family member: 3 (33.3)
Milbury et al. (2015b)	Non-small-cell lung cancer	IA: 3 (30.0) IIIA: 2 (20.0) IIIB: 5 (50.0)	71.22 ± 6.16 (*N* = 10)	5 (35.7)	68.77 ± 5.99 (*N* = 10)	9 (64.3)	Spouse: 9 (90.0); Sibling: 1 (10.0)
Mazanec et al. (2017)	Multiple myeloma	III: 6 (40.0) Other: 9 (60.0)	62.93 (EG: *n* = 7; CG: *n* = 8)	7 (46.7)	54.53 (*N* = 15)	11 (73.3)	Spouse: 10 (66.7)
Milbury et al. (2018)	High-grade glioma	IV: 4 (80.0) Other: 1 (20.0)	51.94 ± 20.20 (*N* = 5)	4 (80.0)	58.16 ± 10.15 (*N* = 5)	3 (60.0)	Spouse: 5 (100)
Milbury et al. (2019)	Neuro-oncology	EG: II: 1 (10.0) III: 1 (10.0) IV: 8 (10.0) WLC: II: 1 (10.0) III: 1 (10.0) IV: 8 (10.0)	EG: 47.91 ± 14.66 (*n* = 10) WLC: 44.73 ± 12.23 (*n* = 10)	EG: 5 (50.0) WLC: 5 (50.0)	EG: 52.36 ± 16.00 (*n* = 10) WLC: 48.27 ± 11.88 (*n* = 10)	EG: 7 (70.0) WLC: 6 (60.0)	EG: Spouse: 6 (60.0) Other family member: 4 (40.0) WLC: Spouse: 5 (50.0) Other family member: 5 (50.0)
McDonnell et al. (2020)	Non-small-cell lung cancer		66.50 ± 5.50 (*N* = 26)	16 (61.5)	60.20 ± 14.10 (*N* = 23)	10 (43.4)	Spouse: 17 (74.0) Other relationship: 6 (26)
Sullivan et al. (2021)	Lung cancer	I: 9 (39.0) III: 5 (22.0) IV: 9 (39.0)	67.90 ± 2.60 (*N* = 23)	9 (61.0)			Spouse: 16 (70.0) Adult child: 5 (22.0) Sibling/friend: 2(8.0)
Thieser et al. (2021)	Breast cancer, Lung cancer, etc.		< 45years: 4 46–55years: 7 56–65years: 10 66–75 years: 13 >75 years: 1 Missing data: 3 (*N* = 38)	30 (78.9)	46–55years: 3 56–65years: 10 66–75 years: 13 Missing data: 2 (*N* = 28)	9 (32.1)	Partners, friends, relatives, etc.

EG, Experimental Group; CG, Control Group; WLC, Wait-list Control Group.

**Table 2:** Characteristics of included interventions in reviewed studies.

**References**	**Design**	**Instrument**	**Intervention**	**Outcomes**
Milbury et al. (2015a)	Pre- and post-test designs	Brief fatigue inventory	Contents: Yoga (1) Joint loosening with breath synchronization; (2) Postures and a deep relaxation technique; (3) Breath energization with sound resonance; (4) Meditation. Frequency: 2–3 times/week; 60 min/time Duration: 15 times	Fatigue Survivors: pre- (3.79 ± 2.16) post- (3.98 ± 2.57) *P* = 0.72 Caregivers: pre- (2.46 ± 2.15) post- (2.31 ± 2.24) *P* = 0.81
Milbury et al. (2015b)	Pre- and post-test designs	Brief fatigue inventory	Contents: Tibetan yoga (1) Deep breathing awareness with visualization; (2) Breath retention exercises; (3) Mindfulness and focused attention through guided meditation; (4) Tsa Lung movements; (5) A brief compassion-based meditation. Frequency: 2–3 times/week; 45–60 min/time Duration: 5–6 weeks	Fatigue Survivors: pre- (3.19 ± 2.30) post- (2.72 ± 2.39) *P* = 0.81 Caregivers: pre- (3.72 ± 2.31) post- (2.09 ± 2.34) *P* = 0.03
Mazanec et al. (2017)	RCT (pilot study)	Patient-reported outcomes measurement information system (fatigue) for both patients and caregivers	Contents: EG: Component 1: Psycho-educational: Two NCI survivors and caregiver booklets Component 2: Behavioral: A home-based, low-impact walking activity Frequency: 5 times/week; 30 min/time Duration: 12 weeks CG: Psycho-educational: Two NCI survivors and caregiver booklets	Fatigue: EG: Three (43%) survivors improved; one (17%) caregiver improved. CG: Three (50%) survivors improved; four (67%) caregivers improved.
Milbury et al. (2018)	Pre- and post-test designs	Brief fatigue inventory	Contents: Yoga (1) Joint loosening with mindfulness training; (2) Asanas with deep relaxation techniques; (3) Pranayama with sound resonance; (4) Meditation/guided imagery focusing on love and compassion for self and family caregiver and acceptance of change. Frequency: 2–3 times/week; 60 min/time Duration: 5–6 weekly	Fatigue Survivors: pre- (1.49 ± 1.02) post- (1.51 ± 1.64) *P* = 0.98 Caregivers: pre- (1.07 ± 0.73) post- (1.42 ± 1.30) *P* = 0.65
Milbury et al. (2019)	RCT	Brief fatigue inventory	Contents: EG: Yoga (1) Joint loosening with mindfulness training; (2) Postures; (3) Breathing exercises; (4) Meditation/guided imagery; Frequency: 2–3 time/week; 45 min/time Duration: 12 times WLC: Usual care	Fatigue EG: Survivors: pre- (3.76 ± 3.19) post- (3.02 ± 2.12) WLC: Survivors: pre- (3.42 ± 2.84) post- (3.39 ± 2.50) Caregivers: pre- (1.93 ± 1.83) post- (2.18 ± 1.74) Differences between groups: LSM Fatigue Survivors: EG = −0.88; WLC = 0.07; *P* = 0.04; Caregiver: EG = −1.76; WLC = 2.50; *P* = 0.07
McDonnell et al. (2020)	Pre- and post-test designs	FACIT fatigue scale (version 4)	Contents: Mindful hatha yoga Levely 1: sitting yoga poses Levely 2: 2 additional meditations plus sitting, standing, and floor yoga poses Frequency:1 times/week; 2 h/time Duration: 8 weeks	Level 1 (*n* = 37) Fatigue Survivors: pre- 32.32 post- 36.16 *P* = 0.11 Caregivers: pre- 35.50 post- 39.00 *P* = 0.35 Level 2 (*n* = 12) Fatigue Survivors: pre- 38.43 post- 37.14 *P* = 0.86 Caregivers: pre- 39.40 post- 40.80 *P* = 0.87
Sullivan et al. (2021)	Pre- and post- test designs	Patient-reported outcomes measurement information system (fatigue)	Contents: Yoga Duration: 12 weeks	Fatigue: 54% of survivors improved
Thieser et al. (2021)	Pre- and post-test designs	German version of the brief fatigue inventory	Contents: Ballroom dance Frequency: 1 time/week; 90 min/time Duration: 45 weeks	Fatigue: pre- 3.89 post- 3.45 *P* = 0.36

EG, Experimental Group; CG, Control Group; WLC, Wait-list Control Group; FACIT Fatigue Scale (Version 4), Functional Assessment of Chronic Illness Therapy Fatigue Scale (version 4); LSM, Least Squares Mean.

### 2.5. Collating, summarizing, and reporting the results of the selected studies

Based on information extracted, the results are presented in the following.

## 3. Results

### 3.1. Search results and study description

**Figure 1:**
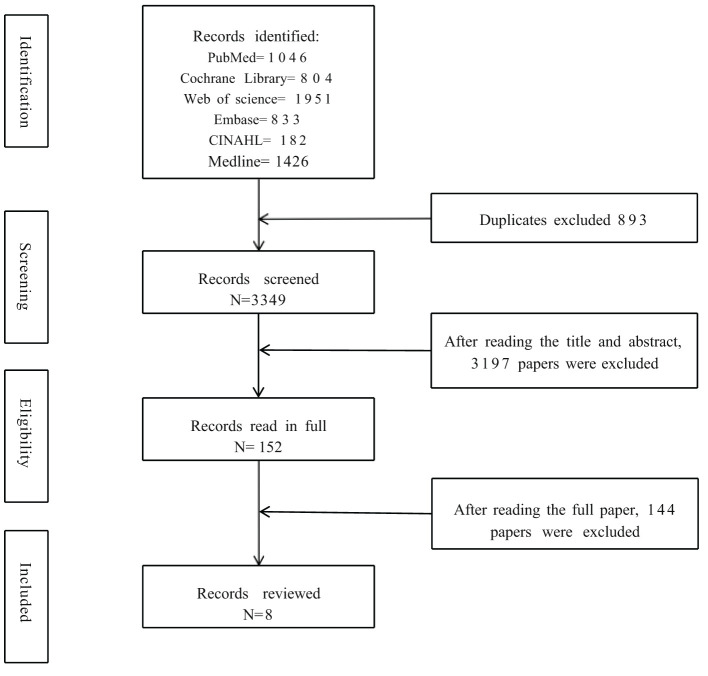
The study flow diagram.

A total of 6,242 articles were identified with 2,893 duplicates eliminated. The remaining 3,349 articles were examined by reading the titles and abstracts. A total of 152 full-text articles were read, and at the end, eight articles met the inclusion criteria. The study selection flowchart is shown in Figure 1. Of the 8 intervention studies, six adopted pre and post-test design and two were randomized controlled trials (RCT). Seven studies were conducted in the United States and one in Germany between the period of 2015 to 2021.

### 3.2. Survivor characteristics

The type of cancer diagnoses included in this study were mainly breast cancer, lung cancer, glioblastoma, and multiple myeloma. The mean age of the survivors ranged from 44.73 to 71.22 years, with more than half of the survivors being female except for one study (Milbury et al., 2015a). Most of the survivors' caregivers were spousal, and a few were adult children, parents and friends. Table 1 summarizes the demographic information of all of the study participants.

### 3.3. Types of intervention

Various forms of intervention described in the literature include yoga, ballroom dancing and walking. The most common form of physical activity was yoga (Milbury et al., 2015a,b, 2018, 2019; McDonnell et al., 2020; Sullivan et al., 2021), which was usually performed under the guidance of a professional yoga instructor and incorporates combined breathing exercises for the chest and abdomen and relaxation techniques for meditation. Ballroom dancing was a two-person, interactive activity that promotes intimacy through physical contact, verbal and non-verbal communication, and physical activity to improve health (Thieser et al., 2021). Walking was a home-based, low-impact walk activity in those included reports. Walking could be a mutual activity either as a dyad or independently, in which the participants were taught to act as “accountability partners” to each other (Mazanec et al., 2017). The duration and frequency of the interventions varied widely among the included studies, ranging from 30 to 120 min in duration, from 1 to 5 times per week, and from 8 to 60 sessions overall.

### 3.4. Findings of reports: CRF

Of the 2 randomized controlled trials included, one showed improvement in CRF and statistically significant differences between groups (Milbury et al., 2019). Another pilot study showed improvement in CRF in 43% of participants in the experimental group, compared to 50% of participants in the control group (Mazanec et al., 2017). In six pre and post-test designs, two of them showed improvement in CRF but the difference was not statistically significant (Milbury et al., 2015b; Thieser et al., 2021), and in addition, one study showed improvement in CRF in 54% of participants (Sullivan et al., 2021). Surprisingly, CRF scores raised in 3 studies, but the differences were not statistically significant (Milbury et al., 2015a, 2018; McDonnell et al., 2020).

## 4. Discussion

The scoping review included eight studies. In two randomized controlled trials, one of the study experimental groups implemented dyadic yoga intervention and the waiting experimental group implemented usual care. There was a significant between-group difference in the improvement of CRF in cancer survivors in the trial group compared to the waiting intervention group. With 10 participants in both the experimental and waiting intervention groups in this trial, the intervention lasted for about 5 weeks with a total duration of 540 min. Both the number of participants and the dose of the intervention were at a low level, and refinements in these two areas may led to more significant results (Milbury et al., 2019). Regarding another RCT study, 7 people in the experimental group underwent a dyadic walking intervention in addition to the control group. The intervention lasted for 12 weeks, with a total intervention time of 1,800 min and a high dose of intervention. CRF improved in 43% (3/7) of survivors in the intervention group and 50% (3/6) of survivors in the control group (Mazanec et al., 2017). The rate of improvement in CRF was smaller in the experimental group than in the control group, a possible explanation for the lack of robustness of the results due to the small sample size. Future studies will need to expand the sample size to compare the outcome variables at baseline and post-intervention and to draw conclusions about whether the intervention is effective.

In six pre and post-test designs, CRF declined in three intervention survivors, with two of the differences not statistically significant (Milbury et al., 2018; Thieser et al., 2021) and the other using a percentage approach to report the percentage of people who improved (Sullivan et al., 2021). The participants in the three studies were 38, 10, and 23 and lasted from 5 to 45 weeks, with a large variation in the dose of the intervention. In the other 3 studies, CRF rose but none of the differences between groups were statistically significant, with 5, 15, and 26 participants completing the intervention, respectively, and total intervention duration ranging from 720 to 960 min (Milbury et al., 2015a, 2018; Sullivan et al., 2021). Cancer survivors of these three pre and post-test designs studies showed a slight increase in CRF, which may be strongly related to the drawbacks of the pre and post-test designs. In addition, the inadequate dose of intervention could be another reason for its lack of efficacy. Based on the trajectory of CRF, it is highly likely that CRF was elevated over the course of the trial, and that remission of the intervention did not offset the elevated CRF, resulting in a non-significant improvement or even an increase in outcome.

Some studies have shown that dyadic-based physical activity interventions can improve CRF in survivors (Milbury et al., 2019). Dyadic interventions tend to be more effective than interventions that focus only on survivors or caregivers (Laver et al., 2017; Poon, 2022), but this scoping review did not conclude. Dyadic-based physical activity intervention increases the contact time of survivors with their partners or other intimate relationships based on the conventional physical activity intervention, which can make survivors feel more support and help from family and society for survivors and caregivers (Milbury et al., 2015a), as well as increase the level of awareness of physical activity. There are studies that reported understanding and support from caregivers reduces the survivor's psychological burden (Samancioglu Baglama and Bakir, 2019), increases the survivor's sense of self-efficacy, increases confidence in overcoming the disease, and allows the survivors to face the disease and life more positively (Ulrich et al., 2021). In addition, having a caregiver accompany the survivor in physical activity largely improved the low compliance in survivor-only physical activity interventions and made it safer for the survivor to exercise with the caregiver (Ellis et al., 2017). Theoretically, dyadic-based physical activity intervention is more effective in improving cancer-related fatigue in cancer survivors, which is worth further exploration in the future.

For dyadic interventions, participant recruitment is a challenge for dyadic-based physical activity interventions (Cheung et al., 2021). Seven of the included studies had fewer than (Sullivan et al., 2021) participants, and small sample sizes can lead to a lack of robustness of results. Recruitment is one of the main problems faced by the dyadic-based physical activity intervention, and most cancer survivors and partners who met the inclusion criteria declined to participate due to time constraints, as well as lack of interest in these programs, treatment needs, psychological stress, and caregiver burden (Milbury et al., 2015b; McDonnell et al., 2020; Sullivan et al., 2021). One study found that time was the biggest barrier to their participation in such programs, and this contributed to the high rate of missed visits in such studies (Ulrich et al., 2021). So we found that in the future, the time and place should be prioritized in the development of dyadic intervention in order to maximize convenience for participants. Previous studies have noted that cancer survivors are more interested in flexible training content and programs (Rudolph et al., 2018; Thieser et al., 2021). To accommodate individual needs, researchers can use modern technology to conduct web-based technical support intervention programs which enable participants to conduct self-help interventions at the appropriate time and place. At the same time, researchers should also consider the form of intervention content, which can be developed in a single form or a combination of multiple forms. Participants can choose their favorite form according to their preferences, so that they can feel more autonomy and awareness of participation in the process of intervention. In order to better improve compliance and participants' interest, phone or SMS reminders and assessments can be timely conducted during the intervention, and participants can be timely compensated and rewarded (Price et al., 2019).

For dyadic-based physical activity interventions aimed at improving CRF, the duration and intensity of the intervention are important. A low dose of the intervention may not be sufficient to improve the survivor's condition, while a high dose of the intervention may increase the survivor's burden and, in turn, contribute to an increase in CRF. Therefore, future studies need to design more rigorous physical activity programs based on guidelines for physical activity in cancer survivors as well as expert consensus. Survivors undergo an exercise risk assessment prior to enrollment to evaluate the possible risks associated with disease, treatment, or comorbidities (Moraitis et al., 2021). In addition, the study design should use a rigorous randomized controlled trial to reduce contamination effects narrowing the differences between groups. And trials need to set an appropriate follow-up period to test the long-term effects of interventions (Milbury et al., 2019). In addition, 0.05 is not appropriate as a criterion for judging statistical differences in the case of small samples, and 0.1 can be used as a criterion for judging the significance of differences, with minimal clinically important differences and other more rigorous methods for judging differences (Lee et al., 2014).

## 5. Study limitations

There are some limitations that must be considered. Four different measurement instruments used in the articles included in this scoping review, and studies did not report standardized scores on the measures administered, making it challenging to compare and interpret results between studies. In addition, only full texts available in English are included, and non-English papers are missed.

## 6. Clinical implications

This scope review summarized and analyzed the status of dyadic-based physical activity intervention, in order to provide reference for clinical medical staff to carry out dyadic-based physical activity intervention program.

## 7. Conclusion

This is the first scoping review examining the outcomes of dyadic-based physical activity interventions to improve CRF. Physical activity is a primary evidence-based intervention strategy for improving CRF. Dyadic-based intervention would also have some additional benefits over a patient-only focused intervention. Theoretically, dyadic-based physical activity intervention has practical implications for cancer survivors. By reviewing the existing literature, we found that dyadic-based physical activity interventions were not do well in improving CRF in different cultural contexts. To contribute to the goal of developing relevant interventions to alleviate cancer-related fatigue. This scoping review collated studies on the impact of dyadic based physical activity interventions on cancer-related fatigue, identified interventions being used in studies, identified gaps, and makes feasibility recommendations for future research.

## Author contributions

DS and YLiu: database searches, screening of titles, study selection, data extraction and collation, and manuscript drafts and editing. CL: manuscript review and language polishing. YLi: methodology, manuscript review, and project administration. All authors contributed to the article and approved the submitted version.

## References

[B1] Arksey H. O'Malley L. (2005). Scoping studies: towards a methodological framework. Int. J. Soc. Res. Methodol. 8, 19–32. 10.1080/1364557032000119616

[B2] Bower J. E. (2014). Cancer-related fatigue–mechanisms, risk factors, and treatments. Nat. Rev. Clin. Oncol. 11, 597–609. 10.1038/nrclinonc.2014.127 25113839 PMC4664449

[B3] Bower J. E. Ganz P. A. Desmond K. A. Rowland J. H. Meyerowitz B. E. Belin T. R.. (2000). Fatigue in breast cancer survivors: occurrence, correlates, and impact on quality of life. J. Clin. Oncol. 18, 743–753. 10.1200/JCO.2000.18.4.743 10673515

[B4] Bower J. E. Wiley J. Petersen L. Irwin M. R. Cole S. W. Ganz P. A.. (2018). Fatigue after breast cancer treatment: biobehavioral predictors of fatigue trajectories. Health Psychol. 37, 1025–1034. 10.1037/hea0000652 30321021 PMC6372460

[B5] Brandão T. Schulz M. S. Matos P. M. (2014). Psychological intervention with couples coping with breast cancer: a systematic review. Psychol. Health. 29, 491–516. 10.1080/08870446.2013.859257 24279379

[B6] Cheung D. S. K. Tang S. K. Ho K. H. M. Jones C. Tse M. M. Y. Kwan R. Y. C.. (2021). Strategies to engage people with dementia and their informal caregivers in dyadic intervention: a scoping review. Geriatr Nurs. 42, 412–420. 10.1016/j.gerinurse.2021.02.002 33639545

[B7] Cobb L. K. Godino J. G. Selvin E. Kucharska-Newton A. Coresh J. Koton S.. (2016). Spousal influence on physical activity in middle-aged and older adults: the ARIC study. Am. J. Epidemiol. 183, 444–451. 10.1093/aje/kwv104 26337074 PMC4772433

[B8] Dean R. (2022). Can improving quality of sleep reduce the symptoms of cancer-related fatigue in adults?: a systematic review. Eur. J. Cancer Care 31, e13597. 10.1111/ecc.13597 35474359 PMC9541520

[B9] Ellis K. R. Janevic M. R. Kershaw T. Caldwell C. H. Janz N. K. Northouse L.. (2017). Engagement in health-promoting behaviors and patient-caregiver interdependence in dyads facing advanced cancer: an exploratory study. J. Behav. Med. 40, 506–519. 10.1007/s10865-016-9819-6 28078502 PMC7286313

[B10] Frikkel J. Gotte M. Beckmann M. Kasper S. Hense J. Teufel M.. (2020). Fatigue, barriers to physical activity and predictors for motivation to exercise in advanced Cancer patients. BMC Palliat. Care 19, 43. 10.1186/s12904-020-00542-z 32234027 PMC7110817

[B11] Haussmann A. Schmidt M. E. Illmann M. L. Schröter M. Hielscher T. Cramer H.. (2022). Meta-analysis of randomized controlled trials on yoga, psychosocial, and mindfulness-based interventions for cancer-related fatigue: what intervention characteristics are related to higher efficacy? Cancers 14, 2016–2038. 10.3390/cancers14082016 35454922 PMC9032769

[B12] Hu Y. Liu T. Li F. (2019). Association between dyadic interventions and outcomes in cancer patients: a meta-analysis. Support. Care Cancer 27, 745–761. 10.1007/s00520-018-4556-8 30604008

[B13] Juvet L. K. Thune I. Elvsaas I. Fors E. A. Lundgren S. Bertheussen G.. (2017). The effect of exercise on fatigue and physical functioning in breast cancer patients during and after treatment and at 6 months follow-up: a meta-analysis. Breast 33, 166–177. 10.1016/j.breast.2017.04.003 28415013

[B14] Laver K. Milte R. Dyer S. Crotty M. (2017). A systematic review and meta-analysis comparing carer focused and dyadic multicomponent interventions for carers of people with dementia. J. Aging Health 29, 1308–1349. 10.1177/0898264316660414 27458254 PMC5680909

[B15] Lee E. C. Whitehead A. L. Jacques R. M. Julious S. A. (2014). The statistical interpretation of pilot trials: should significance thresholds be reconsidered? BMC Med. Res. Methodol. 14, 41–48. 10.1186/1471-2288-14-41 24650044 PMC3994566

[B16] Lin P. J. Kleckner I. R. Loh K. P. Inglis J. E. Peppone L. J. Janelsins M. C.. (2019). Influence of yoga on cancer-related fatigue and on mediational relationships between changes in sleep and cancer-related fatigue: a nationwide, multicenter randomized controlled trial of yoga in cancer survivors. Integr. Cancer Ther. 18, 1534735419855134. 10.1177/1534735419855134 31165647 PMC6552348

[B17] Mazanec S. R. Miano S. Baer L. Campagnaro E. L. Sattar A. Daly B. J.. (2017). A family-centered intervention for the transition to living with multiple myeloma as a chronic illness: a pilot study. Appl. Nurs. Res. 35, 86–89. 10.1016/j.apnr.2017.03.003 28532734

[B18] McDonnell K. K. Gallerani D. G. Newsome B. R. Owens O. L. Beer J. Myren-Bennett A. R.. (2020). A prospective pilot study evaluating feasibility and preliminary effects of breathe easier: a mindfulness-based intervention for survivors of lung cancer and their family members (Dyads). Integr. Cancer Ther. 19, 1534735420969829. 10.1177/1534735420969829 33118443 PMC7604980

[B19] Milbury K. Chaoul A. Engle R. Liao Z. Yang C. Carmack C.. (2015b). Couple-based Tibetan yoga program for lung cancer patients and their caregivers. Psychooncology 24, 117–120. 10.1002/pon.3588 24890852 PMC4437691

[B20] Milbury K. Li J. Weathers S. P. Mallaiah S. Armstrong T. Li Y.. (2019). Pilot randomized, controlled trial of a dyadic yoga program for glioma patients undergoing radiotherapy and their family caregivers. Neuro Oncol. Pract. 6, 311–320. 10.1093/nop/npy052 31386042 PMC6660820

[B21] Milbury K. Mallaiah S. Lopez G. Liao Z. Yang C. Carmack C.. (2015a). Vivekananda yoga program for patients with advanced lung cancer and their family caregivers. Integr. Cancer Ther. 14, 446–451. 10.1177/1534735415583554 25917816 PMC4537807

[B22] Milbury K. Mallaiah S. Mahajan A. Armstrong T. Weathers S. P. Moss K. E.. (2018). Yoga program for high-grade glioma patients undergoing radiotherapy and their family caregivers. Integr. Cancer Ther. 17, 332–336. 10.1177/1534735417689882 28150503 PMC6041937

[B23] Mitchell S. A. Beck S. L. Hood L. E. Moore K. Tanner E. R. (2007). Putting evidence into practice: evidence-based interventions for fatigue during and following cancer and its treatment. Clin. J. Oncol. Nurs. 11, 99–113. 10.1188/07.CJON.99-113 17441401

[B24] Moraitis A. M. Seven M. Sirard J. Walker R. (2021). Expert consensus on physical activity use for young adult cancer survivors' biopsychosocial health: a modified delphi study. J. Adolesc. Young Adult Oncol. 11, 459–469. 10.1089/jayao.2021.0109 34935468

[B25] NCCN (2018). NCCN Guidelines Version 1. 2018 Cancer-Related Fatigue [EB/OL]. Available online at: https://www.nccn.org/professionals/physician_gls/pdf/fatigue.pdf (accessed July 26, 2018).

[B26] Network NCC (2021). NCCN Guidelines: Cancer-Related Fatigue (Version 1.2021). Available online at: https://www.nccn.org/search-result?indexCatalogue=nccnsearch-index&searchQuery=Cancer-Related%20Fatigue&wordsMode=AllWords (accessed January 12, 2023).

[B27] Poon E. (2022). A systematic review and meta-analysis of dyadic psychological interventions for BPSD, quality of life and/or caregiver burden in dementia or MCI. Clin. Gerontol. 45, 777–797. 10.1080/07317115.2019.1694117 31752633

[B28] Price A. Bryson H. Smith A. Mensah F. Goldfeld S. (2019). Processes for engaging and retaining women who are experiencing adversity in longitudinal health services research. BMC Health Serv. Res. 19, 833–844. 10.1186/s12913-019-4698-5 31727073 PMC6854799

[B29] Quinten C. Maringwa J. Gotay C. C. Martinelli F. Coens C. Reeve B. B.. (2011). Patient self-reports of symptoms and clinician ratings as predictors of overall cancer survival. J. Natl. Cancer Inst. 103, 1851–1858. 10.1093/jnci/djr485 22157640 PMC3243678

[B30] Rudolph I. Schmidt T. Wozniak T. Kubin T. Ruetters D. Huebner J.. (2018). Ballroom dancing as physical activity for patients with cancer: a systematic review and report of a pilot project. J. Cancer Res. Clin. Oncol. 144, 759–770. 10.1007/s00432-018-2606-8 29423728 PMC11813434

[B31] Saita E. Ferraris G. Acquati C. Molgora S. Sorge A. Valenti F.. (2022). Dyadic profiles of couples coping with body image concerns after breast cancer: preliminary results of a cluster analysis. Front. Psychol. 13, 869905. 10.3389/fpsyg.2022.869905 35401315 PMC8983958

[B32] Samancioglu Baglama S. Bakir E. (2019). Caregiver-delivered foot reflexology: effects on patients and caregivers. Holist. Nurs. Pract. 33, 338–345. 10.1097/HNP.0000000000000351 31609870

[B33] Sansano-Nadal O. Gine-Garriga M. Brach J. S. Wert D. M. Jerez-Roig J. Guerra-Balic M.. (2019). Exercise-based interventions to enhance long-term sustainability of physical activity in older adults: a systematic review and meta-analysis of randomized clinical trials. Int. J. Environ. Res. Public Health 16, 2527–2541. 10.3390/ijerph16142527 31311165 PMC6678490

[B34] Sharma A. Saneha C. Phligbua W. (2021). Effects of dyadic interventions on quality of life among cancer patients: an integrative review. *Asia Pac. J. Oncol. Nurs*. (2021) 8, 115–131. 10.4103/apjon.apjon_63_20 33688560 PMC7934590

[B35] Stefǎnut A. M. Vintilǎ M. Tudorel O. I. (2020). The relationship of dyadic coping with emotional functioning and quality of the relationship in couples facing cancer-a meta-analysis. Front. Psychol. 11, 594015. 10.3389/fpsyg.2020.594015 33488460 PMC7819877

[B36] Sullivan D. R. Medysky M. E. Tyzik A. L. Dieckmann N. F. Denfeld Q. E. Winters-Stone K.. (2021). Feasibility and potential benefits of partner-supported yoga on psychosocial and physical function among lung cancer patients. Psychooncology 30, 789–793. 10.1002/pon.5628 33452752 PMC8113066

[B37] Tang W. Li Z. Tang C. Wang X. Wang H. (2017). Health literacy and functional exercise adherence in postoperative breast cancer patients. Patient Prefer. Adherence. 11, 781–786. 10.2147/PPA.S127925 28458522 PMC5402901

[B38] Thieser S. Dörfler J. Rudolph I. Wozniak T. Schmidt T. Hübner J.. (2021). Influence of ballroom dancing on fatigue, body image, self-efficacy, and endurance of cancer patients and their partners. Med. Oncol. 38, 15–24. 10.1007/s12032-021-01459-0 33507443 PMC7843482

[B39] Thong M. S. Y. van Noorden C. J. F. Steindorf K. Arndt V. (2020). Cancer-related fatigue: causes and current treatment options. Curr. Treat. Options Oncol. 21, 17–35. 10.1007/s11864-020-0707-5 32025928 PMC8660748

[B40] Tricco A. C. Lillie E. Zarin W. O'Brien K. K. Colquhoun H. Levac D.. (2018). PRISMA extension for scoping reviews (PRISMA-ScR): checklist and explanation. Ann. Intern. Med. 169, 467–473. 10.7326/M18-0850 30178033

[B41] Ulrich G. R. Callan S. Ranby K. W. (2021). Beliefs and interests in physical activity programs of cancer survivors and their romantic partners. J. Cancer Surviv. 1–14. 10.1007/s11764-021-00996-x [Epub ahead of print]. 33595753 PMC7886842

[B42] Winters-Stone K. M. Lyons K. S. Dobek J. Dieckmann N. F. Bennett J. A. Nail L.. (2016). Benefits of partnered strength training for prostate cancer survivors and spouses: results from a randomized controlled trial of the Exercising Together project. J. Cancer Surviv. 10, 633–644. 10.1007/s11764-015-0509-0 26715587

[B43] Wu C. Zheng Y. Duan Y. Lai X. Cui S. Xu N.. (2019). nonpharmacological interventions for cancer-related fatigue: a systematic review and bayesian network meta-analysis. Worldviews Evid. Based Nurs. 16, 102–110. 10.1111/wvn.12352 30919569

[B44] Yang S. Chu S. Gao Y. Ai Q. Liu Y. Li X.. (2019). A narrative review of cancer-related fatigue (CRF) and its possible pathogenesis. Cells 8, 738–756. 10.3390/cells8070738 31323874 PMC6679212

